# Antimicrobial activity of pure platelet-rich plasma against microorganisms isolated from oral cavity

**DOI:** 10.1186/1471-2180-13-47

**Published:** 2013-02-25

**Authors:** Lorenzo Drago, Monica Bortolin, Christian Vassena, Silvio Taschieri, Massimo Del Fabbro

**Affiliations:** 1 Laboratory of Clinical Chemistry and Microbiology, I.R.C.C.S. Galeazzi Orthopaedic Institute, Via R. Galeazzi 4, Milan, 20161, Italy; 2 Department of Biomedical Sciences for Health, University of Milan, Milan, Italy; 3 Department of Biomedical, Surgical and Dental Sciences, I.R.C.C.S. Galeazzi Orthopaedic Institute, University of Milan, Milan, 20161, Italy

**Keywords:** Platelet concentrate, Oral infection, Antimicrobial effect, Minimum inhibitory concentration, Oral cavity

## Abstract

**Background:**

Autologous platelet concentrates (PCs) have been extensively used in a variety of medical fields to promote soft and hard tissue regeneration. The significance behind their use lies in the abundance of growth factors in platelets α-granules that promotes wound healing. In addition, antibacterial properties of PCs against various bacteria have been recently pointed out. In this study, the antimicrobial effect of pure platelet-rich plasma (P-PRP) was evaluated against oral cavity microorganisms such as *Enterococcus faecalis*, *Candida albicans*, *Streptococcus agalactiae*, *Streptococcus oralis* and *Pseudomonas aeruginosa*. Blood samples were obtained from 17 patients who underwent oral surgery procedures involving the use of P-PRP. The antibacterial activity of P-PRP, evaluated as the minimum inhibitory concentration (MIC), was determined through the microdilution twofold serial method.

**Results:**

P-PRP inhibited the growth of *Enterococcus faecalis*, *Candida albicans*, *Streptococcus agalactiae* and *Streptococcus oralis*, but not of *Pseudomonas aeruginosa* strains.

**Conclusions:**

P-PRP is a potentially useful substance in the fight against postoperative infections. This might represent a valuable property in adjunct to the enhancement of tissue regeneration.

## Background

In the past 20 years, the use of autologous platelet concentrates (PCs) has gained great popularity in a variety of medical fields such as dentistry, oral surgery, orthopedics, sports medicine, dermatology, ophthalmology, cosmetic and plastic surgery. The rationale for their use stems from the fact that platelets store and release, upon activation, growth factors such as PDGF, TGF-β, EGF, VEGF, IGF-1, FGF, HGF and other molecules that modulate the wound healing response in both hard and soft tissues. In addition, anti-inflammatory properties of PCs have been pointed out associated with a marked reduction of postoperative pain and swelling [1-3].

Recently, the clinical and *in vitro* antibacterial effect of human PCs has been reported against bacteria such as *Staphylococcus aureus*, *Staphylococcus epidermidis*, *Escherichia coli* and *Klebsiella pneumoniae* while no activity has been found against *Enterococcus faecalis*, *Pseudomonas aeruginosa*, *Enterobacter cloacae*, *Bacillus cereus* and *Bacillus subtilis*[4-12].

The mechanism of the antibacterial effect of PCs is not yet fully understood. Existing evidence suggests that platelets may play multiple roles in antimicrobial host defense: they generate oxygen metabolites, including superoxide, hydrogen peroxide and hydroxyl free radicals; [13-15] they are capable of binding, aggregating, and internalizing microorganisms, which enhances the clearance of pathogens from the bloodstream; they participate in antibody-dependent cell cytotoxicity functions to kill protozoal pathogens; finally, platelets release an array of potent antimicrobial peptides [16,17].

Several techniques are available for the production of PCs, leading to products with different biological characteristics. The various PCs can be classified into four main categories, depending on their leucocyte and fibrin content: pure platelet-rich plasma (P-PRP), pure platelet-rich fibrin (P-PRF), leukocyte- and platelet-rich plasma (L-PRP) and leukocyte- and platelet-rich fibrin (L-PRF). [18] L-PRP and L-PRF might contain substantial amount of white blood cells. The respective effects of platelets and leucocytes in PCs have not been elucidated yet, and the contribution of leucocytes to the observed overall effect remains unclear [19]. Therefore in this study we decided to use a widely documented technology developed in 1999 by Anitua that allows the production of leukocyte-poor platelet concentrate [20].

The aim of this study was to evaluate *in vitro* the antibacterial effect of P-PRP against microorganisms colonizing the oral cavity such as *Enterococcus faecalis*, *Candida albicans*, *Streptococcus agalactiae*, *Streptococcus oralis* and *Pseudomonas aeruginosa*.

## Methods

### Donors

Blood samples were obtained from 17 adult patients (two men, 15 women; mean age 59 ± 11 years, age range 34–75 years) who underwent oral surgery procedures (dental implant placement, tooth extraction) involving the use of P-PRP. All subjects were in general good health (ASA 1–2). No patient took antibiotics during the month before surgery, nor was under anticoagulant or immunosuppressive therapy. Written informed consent for participation in the study was obtained from all patients. The present research was performed within the guidelines of the Helsinki Declaration for biomedical research involving human subjects. The study was approved by the Review Board of the Galeazzi Orthopedic Institute.

### Blood collection and production of P-PRP

Fresh human whole blood from donors was processed using PRGF® System IV (BTI, Biotechnology Institute, Vitoria, Alava, Spain) to create a platelet concentrate, according to manufacturer’s protocol. Briefly, peripheral blood from each donor was taken by venipuncture into 5 ml blood-collecting tubes with 3,8% (wt/vol) trisodium citrate as anticoagulant. Blood was centrifuged at 460 g for 8 min at room temperature. After centrifugation, 3 components were obtained: red blood cells, a thin layer of leukocytes referred to as “buffy coat” and plasma. The 1 ml plasma fraction located above the red cell fraction, but not including the buffy coat, was collected.

### Determination of platelet and leukocyte count

Platelet concentration in whole blood and P-PRP was counted automatically using a hematology analyzer (Sismex XE-2100, Norderstedt, GER). To evaluate the purity of P-PRP, we have also performed a white blood cells count both in whole blood and P-PRP. According to Anitua et al. [8], leukocyte levels in P-PRP must be lower than in whole blood (< 10^3^/μl).

### Activation of P-PRP

P-PRP was activated shortly before use. In order to initiate clotting and trigger the release of platelet content, CaCl2 was added (50 μl per ml of P-PRP).

### Bacterial strains

Clinical isolates collected from patients with oral and dental infectious diseases have been used.

Microorganisms were stored at −80°C before analysis. In particular, we selected the most representative microorganisms colonizing and affected the oral cavity belonging to gram positive, gram negative and fungi, such as *E*. *faecalis* (3 vancomycin-sensitive *enterococcus* (VSE) and 2 vancomycin-resistant *enterococcus* (VRE)), *C*. *albicans*, *S*. *agalactiae*, *S*. *oralis* and *P*. *aeruginosa*. This strains were previously identified by biochemical identification (API system and Vitek2 Compact, Biomerieux, Marcy l'Etoile, France) and confirmed by DNA sequencing of about 80 pb of variable regions V1 and V3 of the 16S rRNA gene by Pyrosequencing (PSQ96RA, Diatech, Jesi, Italy). For each species, we used five strains isolated from different patients that presented dental abscesses. Each strain presented different characteristics (e.g. different antibiotic resistance). In addition, ATCC strains were used as control: *E*. *faecalis* ATCC #29212, *C*. *albicans* ATCC #928, *S*. *agalactiae* ATCC #13813, *S*. *oralis* ATCC #35037 and *P*. *aeruginosa* ATCC #27853.

Before use, strains were thawed and reconstituted in appropriate medium (e.g. Brain Heart Infusion broth (BHI; Biomerieux, Marcy l'Etoile, France) additioned with 5% defibrinated blood) at 37°C for 24 hours.

### Determination of antibacterial activity

The minimum inhibitory concentration (MIC), defined as the lowest concentration of an antimicrobial substance that will inhibit the visible growth of a microorganism, was determined by broth microdilution method.

After seeding in appropriate medium (Trypticase Soy Agar or Columbia Blood Agar; Biomerieux, Marcy l'Etoile, France), a suspension in BHI was prepared for each strain, with an optical density equal to 0,5 McFarland (1 × 10^8^ CFU/mL). After obtaining a concentration of 1 × 10^4^ CFU/mL using appropriate dilutions, 10 μl of each suspension were inoculated in a 96-wells microplate containing 100 μl of BHI and a serial dilution of activated P-PRP. In order to asses that the bacteria and yeast have not responding in an unexpected manner, for each strain, we have performed a positive control which consists in inoculate the bacterial suspension in BHI without P-PRP. After incubation at 37°C for 24 hours, MIC values were read. MIC values correspond to the concentration of P-PRP present in the last well in which a bacterial growth is observable. The assay was performed in duplicate for each strain and, if the two MIC differed by more than two wells, the assay was repeated. Results were expressed as mean ± standard deviation.

A minimum bactericidal concentration (MBC) test was also performed. MBC is the lowest concentration of a substance required to kill a particular bacterium. It was determined from broth microdilution MIC tests by subculturing 100 μl of bacterial suspension to agar media.

## Results

As expected, the P-PRP produced was leukocyte-depleted (0,34 ± 0,27) × 10^3^/μl. In order to obtain the minimum platelet concentration ranges of P-PRP capable of inhibiting bacterial growth, we calculated the mean MIC of the 5 strains tested for each microorganism.

Values are presented in Table 1. MIC are expressed as number of platelets/μl. As can be seen from the data, the platelet concentration ranges are fairly uniform among microorganisms, except for *C*. *albicans*, whose range of MIC is about twice the others, and for *P*. *aeruginosa*, which is not inhibited by P-PRP. *S*. *oralis* seems to be more sensible than other bacteria to the antibacterial activity of P-PRP. No differences were observed between *E*. *faecalis* VRE and *E*. *faecalis* VSE regarding susceptibility to P-PRP.

**Table 1:** Antibacterial activity of P-PRP against oral microorganisms

**N° of patient**	**MIC (n° platelets/μl)**				
	***E. faecalis *VRE**	***E.faecalis *VSE**	***C. albicans***	***S. agalactiae***	***S. oralis***
1	34.475 ± 13.488	29.550 ± 11.013	88.650 ± 22.025	34.457 ± 13.504	8.618 ± 3.372
2	32.500 ± 19.902	35.750 ± 17.801	117.000 ± 29.069	39.000 ± 14.534	3.250 ± 1.112
3	5.738 ± 2.138	4.303 ± 1.069	61.200 ± 20.950	26.775 ± 10.475	3.346 ± 1.310
4	12.488 ± 3.103	16.650 ± 6.205	49.950 ± 12.410	8.305 ± 3.114	7.650 ± 2.619
5	7.613 ± 5.004	6.831 ± 5.263	112.500 ± 27.951	10.937 ± 4.279	2.734 ± 1.070
6	13.956 ± 6.949	13.956 ± 6.949	81.200 ± 27.797	8.881 ± 3.475	7.612 ± 2.837
7	6.581 ± 1.635	5.850 ± 2.006	210.600 ± 52.324	17.550 ± 6.540	26.325 ± 6.540
8	5.375 ± 3.292	5.913 ± 2.944	68.800 ± 23.552	34.400 ± 11.776	34.400 ± 11.776
9	28.425 ± 10.593	21.319 ± 5.297	75.800 ± 25.948	8.290 ± 3.243	8.290 ± 3.244
10	5.611 ± 2.195	4.809 ± 1.792	38.475 ± 14.339	12.825 ± 4.391	14.428 ± 3.585
11	24.200 ± 8.284	21.175 ± 8.284	108.900 ± 27.056	36.300 ± 13.528	33.275 ± 16.569
12	14.000 ± 4.793	13.125 ± 6.187	31.500 ± 7.826	15.750 ± 3.913	17.500 ± 10.717
13	9.075 ± 4.519	10.725 ± 5.534	39.600 ± 14.758	33.000 ± 20.208	29.700 ± 7.279
14	19.906 ± 11.682	15.641 ± 11.682	68.250 ± 25.435	15.640 ± 7.788	4.976 ± 1.947
15	24.850 ± 9.722	21.300 ± 7.938	63.900 ± 15.876	49.700 ± 19.444	6.212 ± 2.431
16	14.850 ± 10.757	11.550 ± 4.519	46.200 ± 18.075	9.900 ± 3.690	18.150 ± 9.037
17	11.375 ± 5.870	8.750 ± 5.358	86.800 ± 53.677	12.250 ± 4.793	6.125 ± 2.396
**RANGE**	**5.375 – 34.475**	**4.303 – 35.750**	**31.500 – 210.600**	**8.290 – 49.700**	**2.734 – 34.400**

Data are expressed as mean ± standard deviation.

MIC values observed for ATCC bacterial strains fell into the same platelet concentration ranges as those of the corresponding clinical isolates.

MBC tests showed that *C*. *albicans* was never killed by P-PRP, while the other microorganisms were killed at concentrations 3–4 times the MIC.

## Discussion

The regenerative potential of PCs has been explored considerably during the last two decades. On the contrary, in the available literature only few reports can be found about their antimicrobial effects.

To date, the components responsible for the antimicrobial activity of PCs remain poorly understood, in particular because these materials are a complex mixture of platelets, white blood cells and plasma. The respective impact of the plasma and cellular components has not been studied in detailyet. Several antimicrobial factors have been proposed, including platelet antimicrobial proteins and peptides of the innate immune defense, or platelet α-granules components, such as complement and complement-binding proteins. [17,21-26] Direct interaction of platelets with microorganisms and participation in antibody-dependent cell cytotocity and white blood cells in direct bacterial killing, release of myeloperoxidas, activation of the antioxidant responsive element and antigen-specific immune response have also been suggested. [12,15,27] The role of leucocytes within PCs is a matter of intense debate. Some authors have suggested that inclusion of white blood cells in PCs may help to improve the stability of the scaffold and increase the antimicrobial potential. [18] However, Anitua et al. [20] results showed that a further leucocyte dose did not significantly improve the antimicrobial properties of P-PRP. It is also possible that the additional leukocyte content might increase the inflammatory response at the site because of the metalloproteases, pro-inflammatory proteases and acid hydrolases secreted by white blood cells [28].

Bacterial infection is one of the most serious complications impairing wound healing and tissue regeneration. Even when applying strict disinfection, bacteria can infiltrate and colonize the underlying tissues of the wound. The combination of proteolytic enzymes, toxin-rich bacterial exudates and chronic inflammation can alter growth factors and metalloproteinases, thereby affecting the cellular machinery needed for cell proliferation and wound healing [29,30].

Developing approaches and strategies that may help to control or prevent the problem of wound infections would have considerable clinical, social and economic effects.

Our study has shown that P-PRP was active against microorganisms colonizing the oral cavity such as *E*. *faecalis*, *C*. *albicans*, *S*. *agalactiae* and *S*. *oralis*, but not against *P*. *aeruginosa*. Except for *E*. *faecalis* and *P*. *aeruginosa*, PCs have never been tested against such microorganisms.

*E*. *faecalis* is associated with different forms of periradicular disease, including primary extraradicular and post-treatment persistent infections. [31] Such microorganism possesses the ability to survive the effects of root canal treatment and persists as a pathogen in the root canals and dentinal tubules of teeth. Implementing methods to effectively eliminate *E*. *faecalis* from the dental apparatus is a challenge. We found that P-PRP was active at low platelet concentration ranges (1–2 orders of magnitude lower than the baseline blood values) against this microorganism, while Bielecki et al. [10] observed no activity of platelet concentrate. The reasons for this discrepancy may lie in the different protocol used for platelet concentrate production, which can lead to products with different biological characteristics, or in the different sensibility of the method (Kirby-Bauer disc-diffusion method) used to evaluate the susceptibility to platelet concentrate.

Oral candidosis is the most common fungal infection encountered in general dental practice. It manifests in a variety of clinical presentations and can occasionally be refractory to treatment. It is caused by commensal *Candida* species. While a large majority of healthy individuals harbor strains of *Candida* intraorally, only selected groups of individuals develop oral candidosis. The most commonly implicated strain is *C*. *albicans*, which is isolated in over 80% of oral candidal lesions. [32] In the present study, we observed that P-PRP was active against *C*. *albicans* at higher plateletconcentration ranges (same order of magnitude of the baseline blood values) than those effective against the other bacteria tested. This result is consistent with the findings of Tang et al. who tested *in vitro* antimicrobial activity of seven antimicrobial peptides isolated from human platelets, and noticed that they were more potent against bacteria than fungi [17].

*S*. *agalactiae*, *S*. *oralis* and *P*. *aeruginosa* are some of the many oral biofilm bacteria. We observed that P-PRP was active against *S*. *agalactiae* and *S*. *oralis* at platelet concentration ranges similar to the range which inhibited *E*. *faecalis*. On the contrary, we found no activity of P-PRP against *P*. *aeruginosa* at the concentrations used in this experiment. This result is in line with the findings of Bielecki et al. and Burnouf et al., who even observed that platelet concentrate induced growth of this microorganism, suggesting that platelet concentrate may induce a flare-up of infection from *P*. *aeruginosa*. [10,11] The value of PCs in the presence of a co-existing infection with this bacterium is therefore uncertain.

In our study we also used standard ATCC bacterial strains, which may behave in a way different from isolates, in order to assure reliability of results and reproducibility of experimentation. Results were similar to those obtained with clinical isolates of bacteria.

In addition, we performed a MBC test. We found such test difficult to perform, as P-PRP coagulates at high concentrations. We observed that *C*. *albicans* was never killed, while the other microorganisms were killed at concentrations 3–4 times the MIC. Further studies are necessary to investigate the potential bactericidal effect of P-PRP. In this study we tested P-PRP in the formulation commonly used in dentistry and oral surgery (that is, plasma fraction activated with CaCl2 to form a solid coagulum) to assess the potentiality of the use of such preparation in routine clinical practice. Future research may be focused on the analysis of the contribution of individual P-PRP components by employing methods such as separation (e.g. by fractionation according to size) or inactivation (e.g. by exposure to modifying agents, such as specific proteases, or to physical factors, such as heat treatment).

## Conclusions

In conclusion, PCs are safe autologous products, which can be easily prepared during surgery and possess an antibacterial activity. They could be potentially useful substances in the fight against postoperative infections and might represent the linking of osteoinductive and antimicrobial activity. Further research should investigate PCs antimicrobial capacity compared to antibiotics, their exact antibacterial spectrum and prove its efficacy in the *in vivo* situation. The influence of patients’ characteristics (sex, age, hematocrit, platelet count, drug assumption, etc.…) on antimicrobial activity should be also clarified.

## Competing interests

The authors declare that they have no financial or non-financial competing interest.

## Authors’ contributions

LD: Conceived the study, participated in its design and coordination and revised the manuscript. BM: Acquired data, participated in their analysis and interpretation and drafted the manuscript. CV: Acquired data, participated in their analysis and interpretation and drafted the manuscript. ST: Revised the manuscript. MdF: Conceived the study, participated in its design and coordination and revised the manuscript. All authors read and approved the final manuscript.
